# MicroRNA-like viral small RNA from porcine reproductive and respiratory syndrome virus negatively regulates viral replication by targeting the viral nonstructural protein 2

**DOI:** 10.18632/oncotarget.12703

**Published:** 2016-10-17

**Authors:** Na Li, Yunhuan Yan, Angke Zhang, Jiming Gao, Chong Zhang, Xue Wang, Gaopeng Hou, Gaiping Zhang, Jinbu Jia, En-Min Zhou, Shuqi Xiao

**Affiliations:** ^1^ College of Veterinary Medicine, Northwest A&F University, Yangling, Shaanxi 712100, China; ^2^ Experimental Station of Veterinary Pharmacology and Veterinary Biotechnology, Ministry of Agriculture, Yangling, Shaanxi 712100, China; ^3^ College of Animal Science and Veterinary Medicine, Henan Agricultural University, Zhengzhou, Henan 450002, China; ^4^ College of Plant Protection, Northwest A&F University, Yangling, Shaanxi 712100, China; ^5^ State Key Laboratory of Crop Stress Biology for Arid Areas, College of Plant Protection, Northwest A&F University, Yangling, Shaanxi 712100, China

**Keywords:** microRNA, PRRSV, viral small RNAs (vsRNAs), viral replication, autoregulation

## Abstract

Many viruses encode microRNAs (miRNAs) that are small non-coding single-stranded RNAs which play critical roles in virus-host interactions. Porcine reproductive and respiratory syndrome virus (PRRSV) is one of the most economically impactful viruses in the swine industry. The present study sought to determine whether PRRSV encodes miRNAs that could regulate PRRSV replication. Four viral small RNAs (vsRNAs) were mapped to the stem-loop structures in the ORF1a, ORF1b and GP2a regions of the PRRSV genome by bioinformatics prediction and experimental verification. Of these, the structures with the lowest minimum free energy (MFE) values predicted for PRRSV-vsRNA1 corresponded to typical stem-loop, hairpin structures. Inhibition of PRRSV-vsRNA1 function led to significant increases in viral replication. Transfection with PRRSV-vsRNA1 mimics significantly inhibited PRRSV replication in primary porcine alveolar macrophages (PAMs). The time-dependent increase in the abundance of PRRSV-vsRNA1 mirrored the gradual upregulation of PRRSV RNA expression. Knockdown of proteins associated with cellular miRNA biogenesis demonstrated that Drosha and Argonaute (Ago2) are involved in PRRSV-vsRNA1 biogenesis. Moreover, PRRSV-vsRNA1 bound specifically to the nonstructural protein 2 (NSP2)-coding sequence of PRRSV genome RNA. Collectively, the results reveal that PRRSV encodes a functional PRRSV-vsRNA1 which auto-regulates PRRSV replication by directly targeting and suppressing viral NSP2 gene expression. These findings not only provide new insights into the mechanism of the pathogenesis of PRRSV, but also explore a potential avenue for controlling PRRSV infection using viral small RNAs.

## INTRODUCTION

Porcine reproductive and respiratory syndrome (PRRS) is one of the most prevalent viral diseases in swine. Infection progressively leads to reproductive failure in pregnant sows and respiratory distress in young pigs, causing significant economic losses to the swine industry worldwide each year [1–3]. The causative pathogen of PRRS is the PRRS virus (PRRSV), a small enveloped linear, positive-sense single-stranded RNA virus, which is a member of the order *Nidovirales* in the family *Arteriviridae* [4]. The genotypes of known PRRSV strains can be divided into two groups, genotype I viruses (European) and genotype II viruses (American), with most Chinese isolates belonging to the latter group [5–7].

The PRRS epidemic in China was particularly devastating, due to unprecedented large-scale outbreaks of highly pathogenic PRRS (HP-PRRS) in 2006 [3, 8, 9]. The HP-PRRS virus (HP-PRRSV) contains a discontinuous deletion of 30 amino acids within the viral non-structural protein 2 (NSP2). In China, HP-PRRS coexists with the long-established, low pathogenic North American type PRRSV strains. Because these coexistent strains cannot be controlled with the same vaccine, current vaccination strategies cannot effectively control PRRSV infection. Therefore, it is imperative to better understand the mechanisms of PRRSV pathogenesis in order to facilitate the development of more effective control measures.

MicroRNAs (miRNAs) are small (17-24 nucleotides) non-coding single-stranded RNAs which regulate gene expression at the post-transcriptional level by either inducing mRNA degradation or inhibiting mRNA translation. Recent evidence indicates that miRNAs encoded by the host or virus can directly modulate virus replication, as well as alter the host cell response to infection in either a proviral or antiviral manner [10, 11]. Previous reports have shown that several host miRNAs can regulate PRRSV infection using a variety of mechanisms [12, 13]. miR-181 inhibits PRRSV replication by targeting both viral genomic RNA and the receptor CD163 [14, 15]. miR-23 inhibits PRRSV replication by targeting PRRSV RNA [16], while miR-125b reduces PRRSV replication by negatively regulating the NF-κB pathway [17]. miR-26a inhibits PRRSV replication by upregulating type I interferons [18]. Our previous work demonstrated yet another strategy, that miR-24-3p promotes PRRSV replication through suppression of heme oxygenase-1 (HO-1) expression [19].

Viral-encoded miRNAs of other viruses have also been identified and studied. For example, several of the herpesviruses, including Epstein-Barr virus (EBV), Kaposi's sarcoma-associated herpesvirus (KSHV), and Murid herpesvirus 68 (MHV68), encode at least 12 characterized miRNAs, which facilitate infection by suppressing host target genes [20]. miRNAs/viral small RNAs (vsRNAs) generated by viruses with RNA genomes can also regulate viral replication by targeting both host and viral mRNAs, leading to successful infection [21]. Influenza A virus-generated vsRNAs regulate the switch from transcription to replication [22]. West Nile virus (WNV)-encoded KUN-miR-1 facilitates virus replication [23], while a dengue virus (DENV)-encoded vsRNA inhibits viral replication [24].

The majority of animal microRNAs are thought to be generated via sequential cleavage involving nuclear events in which the primary miRNA is cleaved by Drosha, followed by cytoplasmic events where Dicer cuts the pre-miRNA to yield a mature miRNA duplex [25, 26]. While many DNA viruses and retroviruses exploit this canonical miRNA biogenesis pathway to encode miRNAs [27], several cytoplasmic RNA viruses generate functional miRNAs through exploitation of other RNA metabolic activities. Sindbis virus (SINV) generates vsRNAs through cytoplasmic endoribonuclease RNase L cleavage [28]. WNV can be processed in the cytoplasm to generate miRNA by Dicer-dependent non-canonical mechanisms without nuclear involvement of Dicer-1 [23]. Recent research has shown that DENV produces vsRNAs processed by the Ago2 protein [24]. However, it is unknown whether PRRSV encodes miRNAs/vsRNAs. Therefore, we sought to confirm the presence of PRRSV-vsRNAs and elucidate the role that such vsRNAs would play in the progression of PRRSV infection.

In this study, we demonstrate that PRRSV does indeed encode vsRNAs. One vsRNA in particular, PRRSV-vsRNA1, suppresses PRRSV replication by directly targeting the viral nonstructural protein 2 (NSP2) sequence. These data not only provide new insights into the mechanisms of PRRSV pathogenesis, but also support a potential novel strategy to counter PRRSV infection.

## RESULTS

### Prediction and identification of PRRSV-encoded vsRNAs

**Figure 1:**
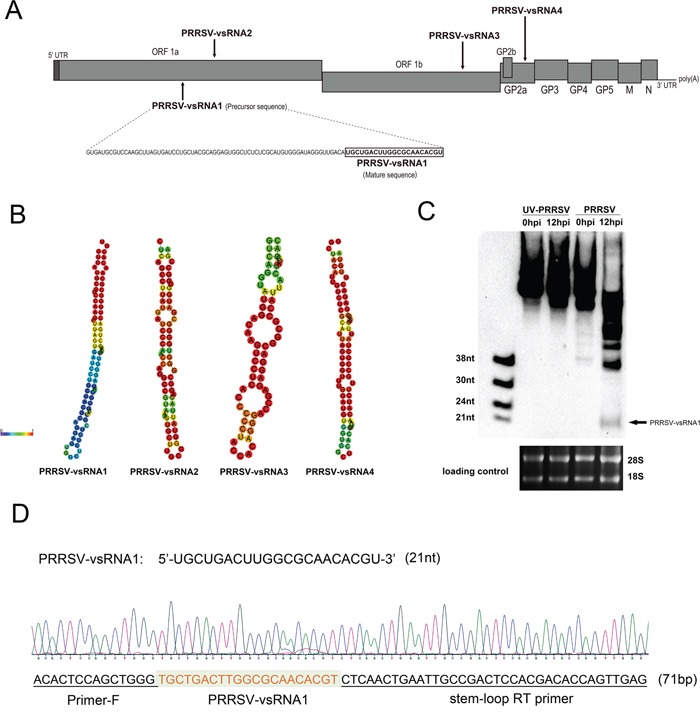
Prediction and identification of PRRSV-encoded miRNA **A.** Schematic diagram of predicted miRNAs in the PRRSV (GD-HD strain) viral genome RNA. **B.** Secondary structure of precursors to PRRSV-vsRNA1-4. **C.** PRRSV-vsRNA1 (black arrow) in mock- or PRRSV-infected PAM cells were detected with digoxigenin-labeled probes containing oligonucleotides complementary to PRRSV-vsRNA1. The 28S and 18S RNA bands stained with ethidium bromide are displayed to demonstrate equal loading. **D.** Cloning validation of the predicted PRRSV-vsRNA1 in PRRSV-infected PAM cells. Sequencing results for the PRRSV-vsRNA1 and primer sequences are underlined in the sequence maps.

Previous bioinformatic analyses have shown that there are four postulated PRRSV miRNAs within the PRRSV genome [29]; however no further experiments have subsequently been performed to confirm their presence or substantial biological functions. In order to identify whether miRNAs could be located in the PRRSV genome, the sequence of the highly pathogenic PRRSV strain, GD-HD, was analyzed using RNAfold and MiPred software. Several candidate sequences were localized to transcriptional regions (Figure 1A) using these tools. PRRSV-vsRNA1 and PRRSV-vsRNA2 were localized to the ORF1a region, PRRSV-vsRNA3 to the ORF1b region, and PRRSV-vsRNA4 to the GP2a region of the PRRSV genome. The minimum free energy (MFE) and free energy of the thermodynamic ensemble for each structure are shown in Table 1. Among these vsRNAs, PRRSV-vsRNA1 exhibited stem-loop structures with the lowest MFE value, −35.4 kcal/mol, which mirrored the stability of a typical hairpin structure (Figure 1B and Table 1). Therefore, PRRSV-vsRNA1 was chosen for further analysis.

**Table 1:** Predicted microRNA in the PRRSV strain GD-HD genome

Predicted microRNA	Mature sequence	Precursor sequence	Minimum free energy prediction (MFE)	Free energy of the thermodynamic ensemble
PRRSV-vsRNA1	UGCUGACUUG GCGCAACACGU	GUGAUGCGUCCA AGCUUAGUGAUC CUGCUACGCAGG AGUGGCUCU CUCGCAUGUGGGA UAGGGUUGACA UGCUGACUUGG CGCAACACGU	−35.40kcal/mol	−36.77 kcal/mol
PRRSV-vsRNA2	GGCGGGUGG GGCGAUGGUGGC	CUCAGCCUUAUGA UCCCAACCAGGCCG UAAAGUGCUUGCGG GUAUUACAGGCGG GUGGGGCGAU GGUGGCCGA	−25.00 kcal/mol	−27.00 kcal/mol
PRRSV-vsRNA3	AGAGGACGGCG CCAUUACUAU	GUCAGGUAUGGACAA GUCCUCACCCCCUAC CACAGGGACCGAGAGG ACGGCGCCAUU ACUAUCGAC	−21.90 kcal/mol	−22.85 kcal/mol
PRRSV-vsRNA4	UCUUGCCGCU AUUGAAGCCGA	GGCUACAUUGUCUCGCAU UAGUGGUUUGGAUGUGGUG GCUCACUUUCAACAUCU UGCCGCUAUUGAA GCCGAGACUUGUA	−28.20 kcal/mol	−29.19 kcal/mol

To investigate whether PRRSV-vsRNA1 is biologically relevant to PRRSV-infected cells, PAM cells were either mock-infected or infected with PRRSV (GD-HD) at an MOI of 1. At 12 hpi, total RNA was analyzed using small RNA northern blot hybridization. A 21 nt small RNA was observed at 12 hpi (Figure 1C) which matched the size of the sequenced small RNA and agreed with results obtained using biological prediction tools. To confirm the identity of the PRRSV-vsRNA1, PRRSV-vsRNA1 from PRRSV (GD-HD)-infected PAM cells was cloned into pMD-18T and the sequence obtained matched the predicted PRRSV-vsRNA1 sequence (Figure 1D).

### Inhibition of PRRSV-vsRNA1 facilitates PRRSV replication

To determine the effect of PRRSV-vsRNA1 on viral RNA replication, PAM cells were separately transfected with either PRRSV-vsRNA1 inhibitor or NC inhibitor. PRRSV-vsRNA1 inhibitor is synthetic oligonucleotides with reverse complementary sequences to the PRRSV-vsRNA1 sequences, while the NC inhibitor was designed based on a random sequence. The viral-specific and NC inhibitors were transfected into cells infected with either of two genotype II PRRSV strains: a highly pathogenic PRRSV strain (GD-HD) or a low pathogenic PRRSV strain (CH-1a) at an MOI of 0.01. The effect on virus biology was assayed in two separate ways. Differences in the relative expression of viral genes and expression levels of viral genomic RNA were determined by qRT-PCR and viral copy number were determined after titration.

**Figure 2:**
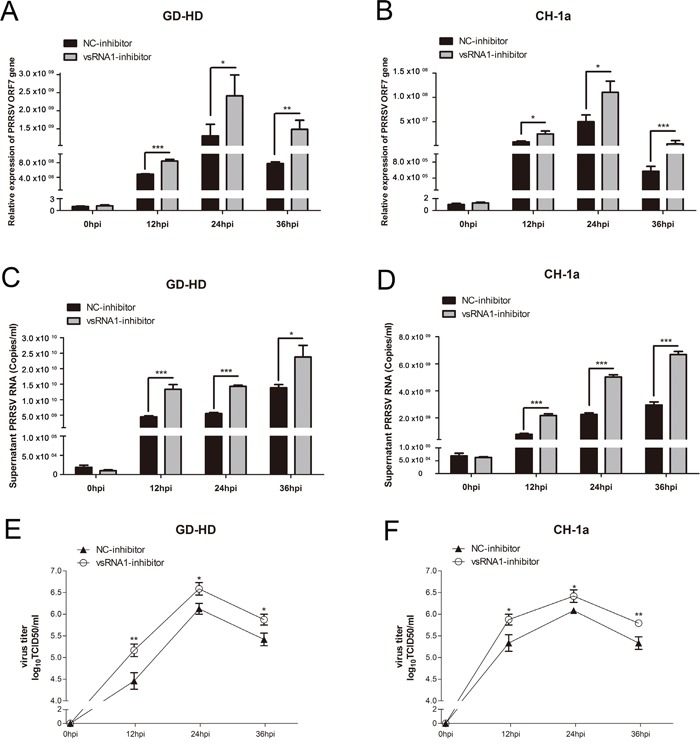
Inhibition of PRRSV-vsRNA1 facilitates PRRSV replication PAM cells were transfected with PRRSV-vsRNA1 inhibitor or NC inhibitor (200 nM) and then infected with either the highly pathogenic PRRSV strain GD-HD (MOI = 0.01) or low pathogenic PRRSV strain CH-1a (MOI = 0.01). **A, B.** Expression levels of PRRSV ORF7 gene in PAM cells were evaluated by qRT-PCR. **C, D.** Number of copies of virus RNA in the supernatant were determined by qRT-PCR. **E, F.** Growth kinetics of PRRSV was evaluated by determination of the TCID_50_. Results are expressed as mean ± SD of three independent experiments. *p <0.05, **p <0.01, ***p < 0.001.

To compare the level of expression of the ORF7 gene and levels of viral genomic RNA, qRT-PCR was performed and demonstrated a higher relative expression of these products in the two PRRSV strains (Figure 2A-2D) in only cells transfected with the PRRSV-vsRNA1 inhibitor from 12 hpi to 36 hpi. This result indicated the possible significance of this small RNA in regulating virus RNA replication. Moreover, viral titers in the supernatants are increased after transfection with PRRSV-vsRNA1 inhibitor, as compared with the NC (Figure 2E-2F).

### PRRSV-vsRNA1 shows strong anti-PRRSV activity

**Figure 3:**
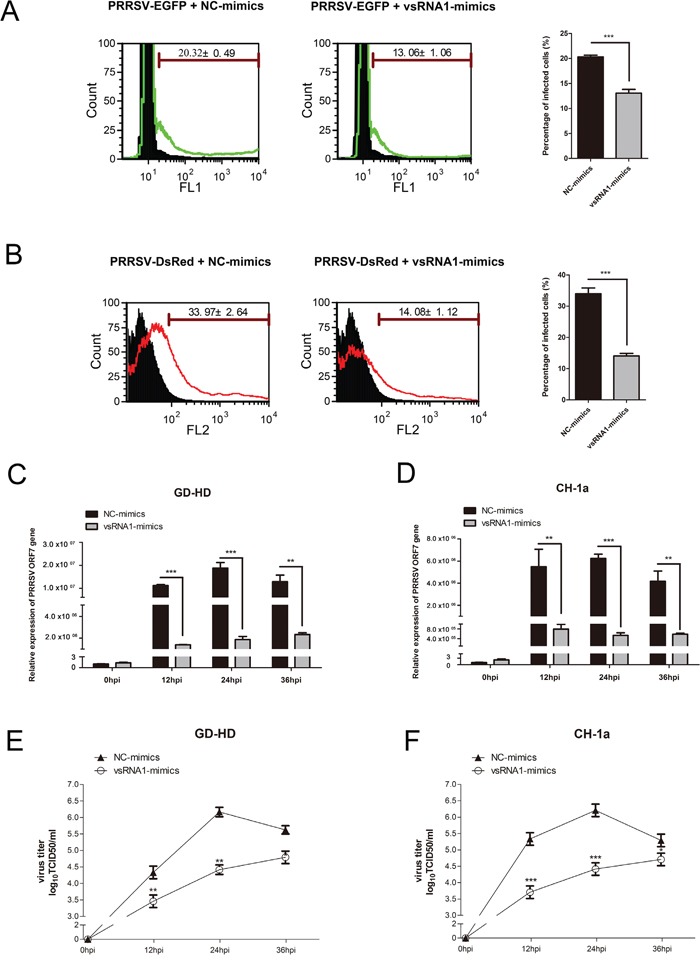
PRRSV-vsRNA1 exhibits antiviral activity in different PRRSV strains *in vitro* MARC-145 cells were transfected with 100 nM of PRRSV-vsRNA1 mimics or NC mimics and then infected with 0.01 MOI of either rHP-PRRSV/SD16/EGFP or rPRRSV/CH-1a/DsRed PRRSV for 48 h. The percentage of EGFP- **A.** or DsRed- **B.** positive cells infected with PRRSV were measured by flow cytometry. PAM cells were transfected with PRRSV-vsRNA1 mimics or NC mimics (100 nM) and then infected with either 0.01 MOI of either highly pathogenic PRRSV strain GD-HD or low pathogenic PRRSV strain CH-1a. Expression levels of PRRSV ORF7 gene in PAM cells was evaluated by qRT-PCR **C, D.** Growth kinetics of PRRSV was evaluated by determination of the TCID_50_ **E, F.** Results are expressed as mean ± SD of three independent experiments. *p <0.05, **p <0.01, ***p < 0.001.

To determine whether PRRSV-vsRNA1 alters PRRSV infection, a recombinant highly pathogenic PRRSV expressing EGFP was used. Quantification of EGFP-positive cells collectively provide a rapid and reliable method to detect the level of PRRSV infection using flow cytometry. MARC-145 cells were transfected with PRRSV-vsRNA1 mimics or NC mimics 24 h before infection with PRRSV strain EGFP-PRRSV at an MOI of 0.01, with subsequent examination of PRRSV-positive cells. As shown in Figure 3A, transfection with PRRSV-vsRNA1 mimics resulted in a 36% decrease of EGFP-PRRSV-positive cells compared with NC mimics-treated cells. To investigate whether the anti-viral activity of PRRSV-vsRNA1 is virus strain-dependent, the same experiment was performed with a recombinant low pathogenic PRRSV expressing DsRed. The result showed a 59% decrease of DsRed-PRRSV-positive cells in cells transfected with PRRSV-vsRNA1 mimics compared with NC mimics-treated cells (Figure 3B). These results indicate that PRRSV-vsRNA1 inhibited virus infection in cells infected with either the HP-PRRSV strain or the low pathogenic PRRSV strain.

To determine if wild-type (WT) PRRSV infection could be prevented by PRRSV-vsRNA1, PAMs were transfected with PRRSV-vsRNA1 mimics or NC mimics 24 h before infection of either GD-HD or CH-1a at an MOI of 0.01. Viral gene expression and viral titers were examined at the indicated timepoints. As shown in Figure 3C and 3D, viral growth of each of the two different PRRSV strains was suppressed significantly in PAM cells transfected with PRRSV-vsRNA1 mimics from 12 hpi to 36 hpi. The expression level of GD-HD ORF7 mRNA decreased by 88%, 90% and 82% at 12, 24 and 36 hpi, respectively, (Figure 3C). Furthermore, the GD-HD viral titers in the supernatants decreased by 0.88, 1.75 and 0.83 log10 at 12, 24 and 36 hpi, respectively, in cells transfected with PRRSV-vsRNA1 mimics compared with NC mimics-treated cells (Figure 3E). The expression level of CH-1a ORF7 mRNA was reduced by 86%, 91% and 86% at 12, 24 and 36 hpi, respectively, (Figure 3D) and the CH-1a viral titers in the supernatants decreased by 1.63, 1.79 and 0.58 log10 at 12, 24 and 36 hpi, respectively, in PRRSV-vsRNA1 mimics-transfected cells compared with NC mimics-treated cells (Figure 3F).

### PRRSV-vsRNA1 inhibits PRRSV replication in a dose-dependent manner

**Figure 4:**
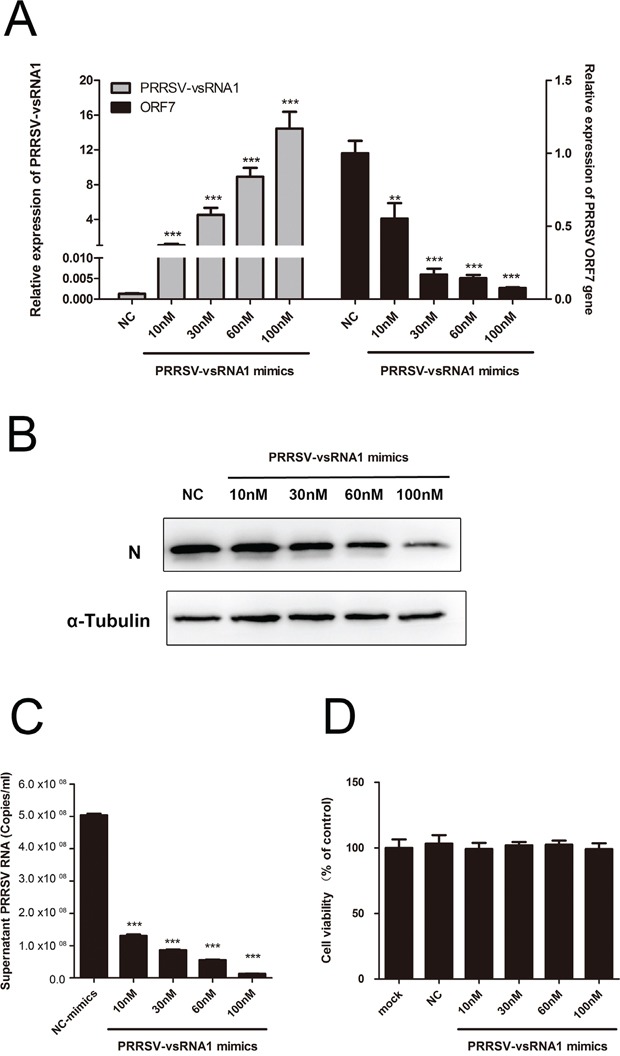
PRRSV-vsRNA1 inhibits PRRSV replication in a dose-dependent manner PAMs were transfected with different concentrations of PRRSV-vsRNA1 mimics followed by infection with GD-HD (MOI = 0.01) for 24 h. Next, the expression levels of PRRSV-vsRNA1 (A left) and ORF7 gene (A right) were assayed by qRT-PCR. **B.** The expression of PRRSV N protein was analyzed by western blot. α-Tubulin was used as an internal control. **C.** Copies of virus RNA in the supernatant were determined by qRT-PCR. **D.** Analysis of the effect of PRRSV-vsRNA1 mimics on the cell viability of PAM cells by MTT assay. Results are expressed as mean ± SD of three independent experiments. **p <0.01, ***p < 0.001.

To further understand the effect of PRRSV-vsRNA1 on PRRSV replication, PAM cells were transfected with increasing concentrations of PRRSV-vsRNA1 mimics before infection with GD-HD at an MOI of 0.01, viral gene expression and virus production were examined at 24 hpi. The dose-dependent increase in PRRSV-vsRNA1 (Figure 4A left) mirrored the reduction in the ORF7 mRNA (Figure 4A right), nonstructural protein NSP2 gene (Supplementary Figure S1) and N protein levels (Figure 4B). PRRSV RNA copies in the supernatant also significantly decreased as the concentration of transfected PRRSV-vsRNA1 mimics increased (Figure 4C). There were no significant cytotoxic effects at the concentrations of PRRSV-vsRNA1 mimics or negative control mimics used (Figure 4D)

### PRRSV-vsRNA1 sequence and expression analyses

**Figure 5:**
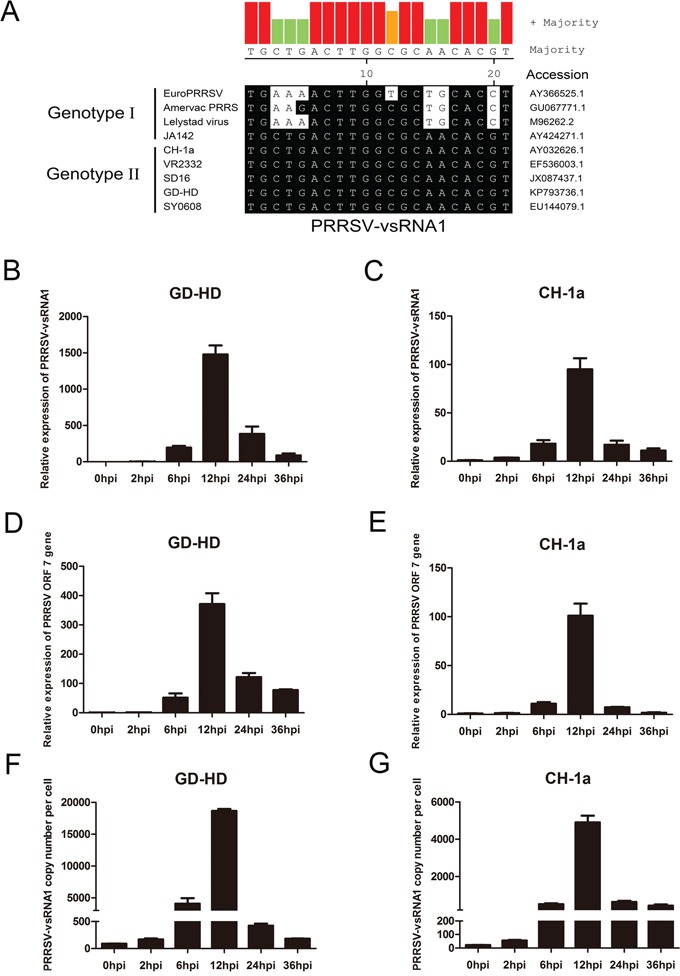
PRRSV-vsRNA1 expression in PRRSV-infected PAMs **A.** Sequence homology of PRRSV-vsRNA1 for the two genotypes of PRRSV. The accession numbers for the nine PRRSV strains are listed. PAM cells were infected with either the highly pathogenic PRRSV strain GD-HD or low pathogenic PRRSV strain CH-1a (MOI = 1). Relative expression levels of PRRSV-vsRNA1 **B, C.** and of the ORF7 gene **D, E.** at indicated timepoints after GD-HD or CH-1a infection were determined by qRT-PCR. To determine expression of PRRSV-vsRNA1 in cells, RNA extracted from various numbers of GD-HD- **F.** or CH-1a- **G.** infected PAM cells at each timepoint was used to determine the copy number of PRRSV-vsRNA1. Chemically synthesized oligonucleotides corresponding to PRRSV-vsRNA1 were used to generate standard curves.

To examine sequence conservation of PRRSV-vsRNA1 in the two PRRSV genotypes, sequence alignment was performed. PRRSV-vsRNA1 was 100% homologous to the PRRSV genotype II sequence and was highly similar to genotype I strains (Figure 5A).

To identify the expression pattern of PRRSV-vsRNA1 during PRRSV infection, we detected PRRSV-vsRNA1 expression in PAMs infected with two genotype II PRRSV strains: GD-HD or CH-1a, each at an MOI of 1. The expression level of PRRSV ORF7 mRNA and PRRSV-vsRNA1 were analyzed from PRRSV-infected PAM cells at 0, 2, 6, 12, 24 and 36 hpi by qRT-PCR. As shown in Figure 5B and 5C, PRRSV-vsRNA1 was upregulated gradually in PRRSV-infected cells and reached its peak expression at 12 hpi. The time-dependent increase in the abundance of PRRSV-vsRNA1 mirrored the upregulation pattern for PRRSV ORF7 RNA (Figure 5D, 5E).

To investigate the copy number of PRRSV-vsRNA1 produced in a single PAM cell, the levels of vsRNA1 in infected PAM cells were quantified by comparing them with known amounts of synthetic PRRSV-vsRNA1 mimics. As shown in Figure 5F, the copy number of PRRSV-vsRNA1 in a single PAM cell infected with the GD-HD strain increased from 88 at 0 hpi to 18,660 at 12 hpi. Moreover, the copy number of PRRSV-vsRNA1 in a single PAM cell infected with CH-1a increased from 22 at 0 hpi to 4,899 at 12 hpi (Figure 5G).

### Drosha and Ago2 are involved in PRRSV-vsRNA1 biogenesis

**Figure 6:**
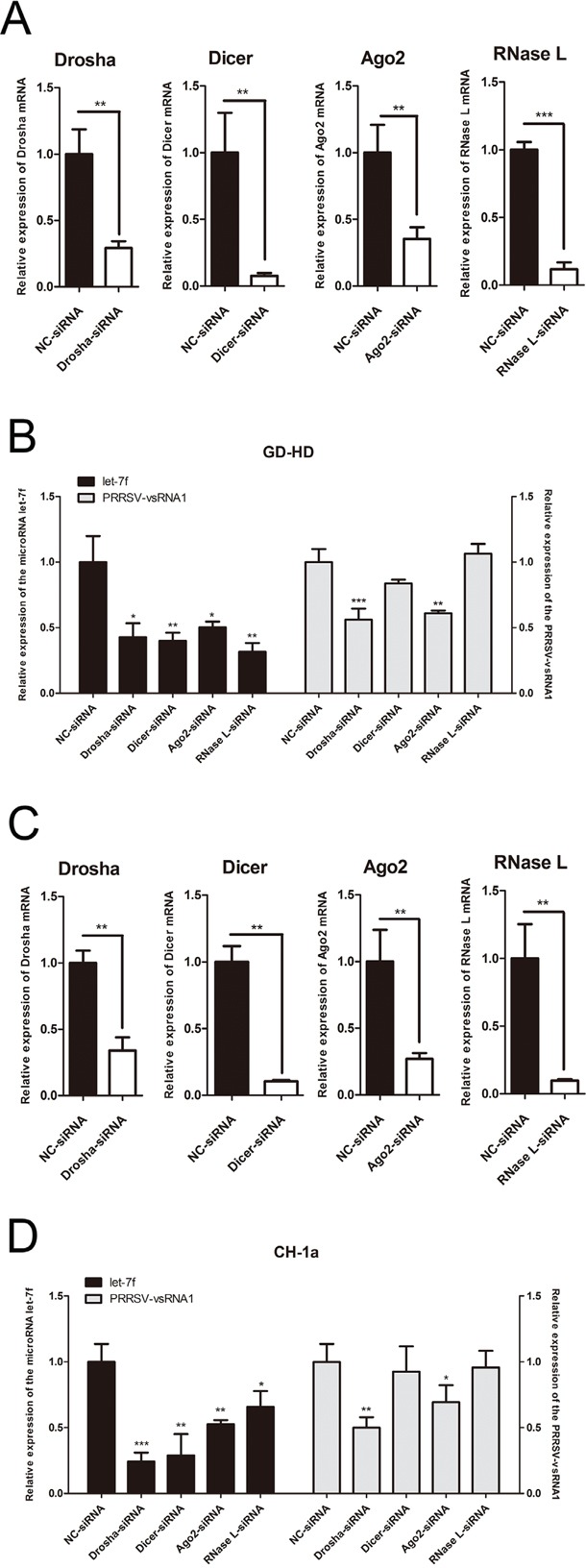
Drosha and Ago2 are involved in the PRRSV-vsRNA1 biogenesis MARC-145 cells were transfected with Drosha/Dicer/Ago2/RNase L siRNAs (50 nM) and then infected with either highly pathogenic PRRSV strain GD-HD (MOI = 1) or low pathogenic PRRSV strain CH-1a (MOI = 1). Relative expression levels of cellular genes Drosha/Dicer/Ago2/RNase L, let-7f and PRRSV-vsRNA1 at 12 hpi after GD-HD **A, B.** or CH-1a **C, D.** infection were determined by qRT-PCR. Results are expressed as mean ± SD of three independent experiments. *p <0.05, **p <0.01, ***p < 0.001.

To find out which protein members of known microRNA biogenesis pathways are involved in the processing of mature PRRSV-vsRNA1, Drosha, Dicer, Ago2 and RNase L genes were silenced in MARC-145 cells then followed by infection of either of two PRRSV strains (GD-HD, CH-1a) at an MOI of 1. Gene expression levels of Drosha, Dicer, Ago2 and RNase L at 12 hpi were reduced dramatically after transfection with gene-specific siRNAs as compared with NC siRNA-transfected cells (Figure 6A, 6C). As a positive control, the expression level of cellular microRNA let-7f was decreased after four of the cellular genes knockdown (Figure 6B left, 6D left). However, only Drosha and Ago2 suppressed biogenesis of mature PRRSV-vsRNA1. The expression levels of mature PRRSV-vsRNA1 in GD-HD-infected and CH-1a-infected Drosha-silenced cells decreased by 43.9% and 50%, respectively; PRRSV-vsRNA1 expression levels in GD-HD-infected and CH-1a-infected Ago2-silenced cells decreased by 39% and 30.6%, respectively. These results suggest the possible role of Drosha and Ago2 in the biogenesis of PRRSV-vsRNA1 (Figure 6B right, 6D right).

### PRRSV-vsRNA1 directly targets the NSP2 sequence within PRRSV genomic RNA

**Figure 7:**
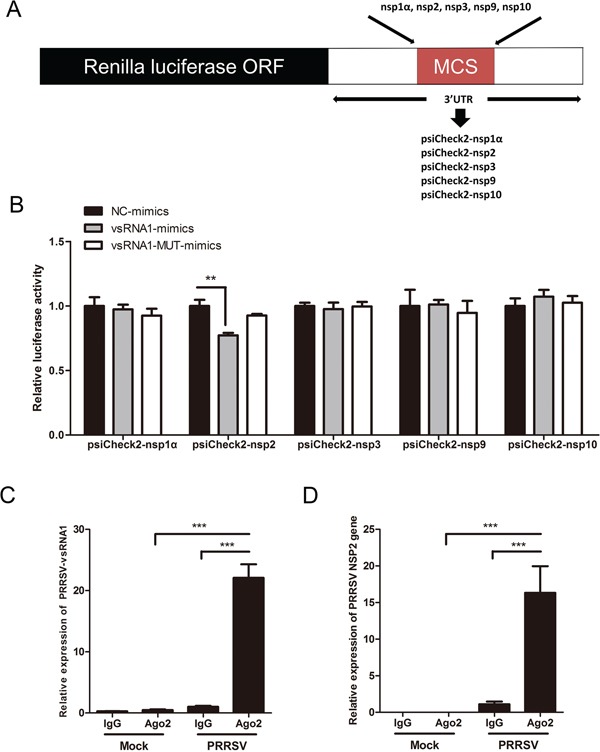
PRRSV-vsRNA1 targets PRRSV genomic RNA **A.** The 5 predicted PRRSV genome binding regions of PRRSV-vsRNA1 were cloned into the 3′UTR-luciferase reporter gene of psiCheck-2. **B.** Luciferase reporter plasmids psiCheck2-nsp1α, psiCheck2-nsp2, psiCheck2-nsp3, psiCheck2-nsp9, or psiCheck2-nsp10 were co-transfected with PRRSV-vsRNA1 mimics or NC mimics. Reporter activities were determined at 36 h post-transfection by dual-luciferase assays. **C, D.** RNA of mock- and GD-HD-infected PAM cells were subjected to Ago2-IP and the relative expression levels of PRRSV-vsRNA1(C) and NSP2 gene (D) in the immunoprecipitates were determined by qRT-PCR. As a negative control, immunoprecipitation was performed using IgG beads. **p <0.01, ***p < 0.001.

Given the inhibitory effect of transfection with PRRSV-vsRNA1 mimics on PRRSV replication, we investigated whether any direct interaction between PRRSV-vsRNA1 and PRRSV genomic RNA exists. First, PRRSV genomic RNA was analyzed for potential PRRSV-vsRNA1 target sequences using RNAhybrid software. Five regions (NSP1α, NSP2, NSP3, NSP9 and NSP10) of PRRSV genomic RNA revealed strong binding sites with MFE values ≤-25 kcal/mol and complete complementarity to the seed region. Next, we created five reporter constructs containing the predicted target site regions: psiCheck2-nsp1α, psiCheck2-nsp2, psiCheck2-nsp3, psiCheck2-nsp9 and psiCheck2-nsp10 (Figure 7A). The effect of PRRSV-vsRNA1 on reporter gene expression was monitored by co-transfection of cells with PRRSV-vsRNA1 mimics and the various luciferase reporter constructs. As shown in Figure 7B, transfection with PRRSV-vsRNA1 mimics significantly decreased luciferase expression by 23% in cells transfected with psiCheck2-nsp2 only, but decreased luciferase was not observed for any of the other four luciferase reporter constructs. Co-transfection with NC mimics and PRRSV-vsRNA1-MUT mimics had no effect on any of the five luciferase reporter constructs.

To further demonstrate the direct interaction between PRRSV-vsRNA1 and the NSP2 gene, immunoprecipitation (IP) analyses were carried out using anti-Ago2 antibody treatment of cell lysates. PAM cells were either mock-infected or infected with the GD-HD strain at an MOI of 0.1 at 12 hpi. RNAs within lysates from PRRSV-infected or non-infected PAM cells were immunoprecipitated using anti-Ago2 monoclonal antibody. Next, the PRRSV-vsRNA1 and NSP2 gene in the co-precipitates were detected by qRT-PCR. As shown in Figure 7C, the PRRSV-vsRNA1 level associated with Ago2 protein was increased by 21.7-fold compared to that of the IgG isotype control. However, the abundance of NSP2 gene increased by approximately 14.8-fold compared to that of the IgG isotype control (Figure 7D). These results demonstrate that PRRSV-vsRNA1 directly binding to the NSP2 region of PRRSV genome in the RNA-induced silencing complex (RISC). Therefore, NSP2 was chosen for further validation studies.

**Figure 8:**
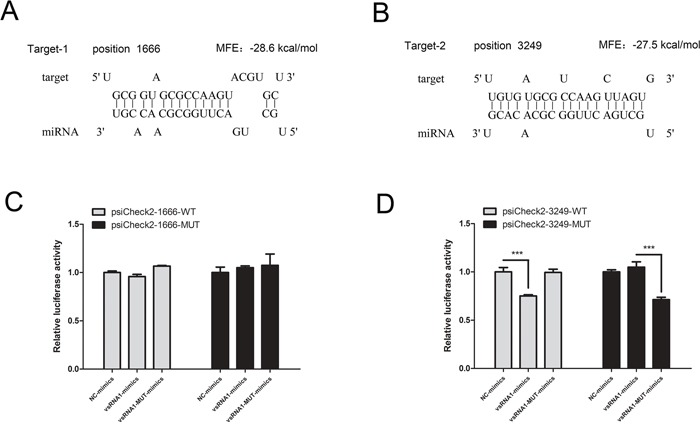
PRRSV-vsRNA1 specifically binds to the PRRSV NSP2 region **A, B.** Two seed-matched target sites for PRRSV-vsRNA1 were predicted within the PRRSV NSP2 region. **C, D.** WT or MUT reporter plasmids, psiCheck2-1666 or 3249, were co-transfected with the PRRSV-vsRNA1 mimics, PRRSV-vsRNA1-MUT mimics, or NC mimics. Reporter activities were determined 36 h post-transfection by dual-luciferase assays. ***p < 0.001.

Two strong binding sites in NSP2, each with an MFE of <-25 kcal/mol and perfect complementarity to the seed region, were predicted by RNAhybrid software at nucleotide positions 1,666 and 3,249 in the PRRSV genome (Figure 8A-8B). To investigate whether PRRSV-vsRNA1 directly targets NSP2 within PRRSV genomic RNA, the WT target sequences and mutated target sequences were cloned into the luciferase reporter vector. As shown in Figure 8C and 8D, PRRSV-vsRNA1 mimics significantly inhibited the luciferase activity of psiCheck2-3249-WT by 25% but did not inhibit psiCheck2-1666-WT luciferase activity. However, disruption of the seed sequence in plasmid psiCheck2-3249-MUT ablated the ability of PRRSV-vsRNA1 mimics to decrease luciferase expression. Similarly, vsRNA1-MUT-mimics did not inhibit the luciferase expression from psiCheck2-1666-WT or psiCheck2-3249-WT. However, the sequence homologous to vsRNA1-MUT-mimics significantly decreased the luciferase activity of psiCheck2-3249-Mut (Figure 8C-8D). These results demonstrate that PRRSV-vsRNA1 can directly target the NSP2 of PRRSV genomic RNA in a sequence-specific manner.

As PRRSV-vsRNA1 was 100% homologous to the PRRSV genotype II sequence, the PRRSV-vsRNA1 target sequences alignment was performed in 19 representative genotype II PRRSV strains, and the results show that target region was conserved in the most genotype II PRRSV strains (Supplementary Figure S2)

## DISCUSSION

Viruses are intracellular pathogens which are unable to carry the large amount of genetic information needed to establish a favorable cellular environment to autonomously synthesize viral proteins and RNA [30]. Subsequently, virus-encoded miRNAs, which occupy only a small space in the viral genome, have evolved rapidly to act on new targets while maintaining low immunogenicity. These properties of viral miRNAs allow viruses to employ a unique strategy for the modulation of both host and viral gene expression necessary to maintain virus viability [27]. So far, more than 500 virus-encoded miRNAs have been identified from both DNA and RNA viruses and are listed on miRBase [31].

In the present study, we identified and characterized miRNA-like small RNAs encoded by PRRSV using bioinformatic prediction and experimental verification tools. Four vsRNAs mapped to the stem-loop structures in the ORF1a, ORF1b and GP2a regions of the PRRSV genome (Figure 1A). Of these, PRRSV-vsRNA1 stem-loop structures exhibited the lowest MFE values, mirroring the stability of a typical hairpin structure (Figure 1B). Northern blot hybridization with a PRRSV-vsRNA1 probe produced distinct bands which matched the size of the sequenced small RNA obtained from PRRSV-infected cells (Figure 1C). Inhibition of PRRSV-vsRNA1 facilitated PRRSV replication (Figure 2). PRRSV-vsRNA1 induction correlated with increased PRRSV RNA expression (Figure 5), which implies that there is a positive correlation between the expression of PRRSV-vsRNA1 and viral RNA during PRRSV infection.

Virus-encoded miRNAs/vsRNAs can regulate viral infection in a variety of ways, including inhibition of the host antiviral immune response, inhibition of viral transcription to regulate the virus life cycle, or promotion of cell survival to support continued viral replication [11]. WNV encodes a vsRNA in its 3′UTR that facilitates virus replication by targeting and increasing the accumulation of GATA4 mRNA in mosquito cells [23]. GATA4 is a zinc-finger transcription factor which involve in lipid trafficking and immune recognition [32]. DENV-vsRNA5 inhibits DENV replication by targeting the viral nonstructural protein 1 sequence [24]. Moreover, our results show that PRRSV-vsRNA1 can inhibit viral replication of the North American PRRSV strains (Figure 3 and Figure 4). This result paves the way for the possible utilization of small RNAs as a treatment option to interfere with North American PRRSV infection.

Persistent infection is a main feature of PRRSV, with infection in some individual pigs lasting up to 251 days [33, 34]. It has been speculated that the inhibition of PRRSV replication by PRRSV-vsRNA1 may actually be associated with persistent PRRSV infection. PRRSV may employ PRRSV-vsRNA1 to auto-regulate production of viral transcripts and orchestrate necessary viral protein expression patterns to establish persistent infection. Another report has shown that MHV68-encoded miRNAs are important to establishing lifelong infection, as they facilitate viral latency within the key virus reservoir of memory B cells [20]. This evidence supports our theory regarding the role of PRRSV-vsRNA1 in persistent viral infection [35, 36].

To investigate whether PRRSV-vsRNA1 was produced through the microRNA canonical biogenesis pathway, RNase III enzymes Drosha and Dicer, which are two important proteins in this pathway, were knocked down. Silencing of Dicer had no effect on PRRSV-vsRNA1 levels. However, silencing of Drosha significantly reduced PRRSV-vsRNA1 levels, suggesting that Drosha may function in the processing of this small RNA. Many studies have demonstrated that cytoplasmic RNA virus replication would not produce viral miRNAs through the canonical biogenesis pathway, due to inaccessibility of the genomic RNA to the endogenous ribonuclease Drosha [37]. However, recent studies have showed that a noncanonical microprocessor containing Drosha and pri-miRNAs is distributed in the cytoplasm in virus-infected cells. Moreover, cytoplasmic Drosha is involved in processing RNA virus-derived pri-miRNAs [38], which suggests PRRSV, a cytoplasmic RNA virus, may utilize the cellular miRNA processing machinery to produce vsRNAs. Furthermore, a putative non-canonical Ago2-dependent or RNase L-dependent pathway has been reported to generate functional small RNA from a cytoplasmic RNA virus [24, 28]. To investigate whether Ago2 or RNase L are involved in PRRSV-vsRNA1 generation, PRRSV-vsRNA1 expression was quantified in Ago2 and RNase L-knockdown experiments using cells infected with two strains of PRRSV. PRRSV-vsRNA1 was downregulated in Ago2-knockdown cells, but not RNase L-knockdown cells. This result suggests that Ago2 is also involved in vsRNA1 generation during PRRSV infection (Figure 6).

MicroRNA-mediated gene regulation can be achieved through partial complementarity between the miRNA recognition element (MRE) and as few as six or seven nucleotides (the seed region) at the 5′-end of the miRNA [39–41]. Additionally, miRNAs can bind to different MREs to regulate gene expression [42]. Our study found that PRRSV-vsRNA1 directly targets the RNA of NSP2 (Figure 7, Figure 8). PRRSV NSP2 is recognized as an immunodominant target of the adaptive immune response [43] and the central region of NSP2 has been shown to take part in the regulation of innate immune evasion [44, 45]. NSP2 is the largest and most genetically diverse protein of the PRRSV NSPs [46]. There may be significant selective pressures favoring divergent NSP2 genetic or protein content [44, 47, 48]. Interestingly, the nucleotide sequence of PRRSV-vsRNA1 is well conserved across all North American (genotype II) PRRSV strains (Figure 5A), signifying existence of evolutionary pressure to maintain the integrity of the sequence within this region. This data supports the idea that PRRSV-vsRNA1 may have a conserved functional role in modulating the expression of its targets. The PRRSV-vsRNA1 targeting sites (nt 3,249 of GD-HD) have been verified within the NSP2 region (Figure 8). PRRSV-vsRNA1 may target the NSP2 region in order to partially inhibit virus replication, avoiding virus over-replication and premature death of host cells.

In summary, we demonstrate for the first time that PRRSV utilizes cellular miRNA processing machinery to encode functional microRNA-like vsRNAs. PRRSV-vsRNA1 negatively regulates PRRSV replication by directly targeting the viral NSP2 sequence. These results provide new insights into the mechanisms of PRRSV pathogenesis and also introduce the possibility of utilizing vsRNAs as a way to control PRRSV infection.

## MATERIALS AND METHODS

### Cells and virus

The PRRSV-permissive monkey kidney cell line MARC-145 was cultured in Dulbecco's modified Eagle's medium (DMEM, Life Technologies) supplemented with 10% fetal bovine serum (FBS) and penicillin-streptomycin (100 U/mL). Porcine alveolar macrophages (PAMs) were isolated from healthy 6-week-old crossbred (Landrace × Yorkshire) weaned PRRSV-negative pigs using a lung lavage technique as previously described [49] with minor modifications [50]. All animal work was performed in strict accordance with the guidelines of, and approval by, the Animal Care and Use Committee of Northwest A&F University.

The following PRRSV strains were used: GD-HD (a highly pathogenic PRRSV strain isolated in the Guangdong Province of China, GenBank ID: KP793736.1) and CH-1a (a low pathogenic PRRSV type 2 strain isolated in China with a high homology to VR-2332, GenBank ID: AY032626). Additionally, two recombinant PRRSVs were used in this study. One strain was constructed on the genetic background of SD16 (a highly pathogenic PRRSV strain isolated in the Shandong Province of China, GenBank ID: JX087437) and engineered to express enhanced green fluorescent protein (EGFP) as an additional ORF (rHP-PRRSV/SD16/TRS6-EGFP). The other strain was constructed on the genetic background of Ch-1R (a low pathogenic PRRSV type 2 strain isolated in China, GenBank ID: EU807840.1) and engineered to express *Discosoma* sp. red fluorescent protein (DsRed) as an additional ORF (rPRRSV/Ch-1R/TRS6-DsRed).

### Viral infection with transfection of microRNA mimics or inhibitor

MicroRNA mimics and inhibitor were synthesized by GenePharma (Shanghai, China). The sequences are shown in Table 3. PAMs were cultured in 24-well plates for 12 h then transfected with PRRSV-vsRNA1 mimics, negative control (NC) mimics, PRRSV-vsRNA1 inhibitor, or NC inhibitor using X-tremeGENE siRNA Transfection Reagent (Roche) according to the manufacturer's instructions. After transfection, cells were infected with PRRSV, supernatants were collected for virus titration or calculation of copy number and cells were collected for RNA quantification or western blot analysis.

### MTT assay

The cell viability of PAMs with microRNA mimics was evaluated by MTT [3-(4,5-Dimethyl-2-thiazolyl)-2,5-diphenyl-2H-tetrazolium bromide] assay. PAMs in 96-well plates were transfected with specific concentration PRRSV-vsRNA1 mimics or negative control mimics and cultured for 48 h. Then 20 μL MTT solution (5 mg/ml) (Sigma-Aldrich) was added to each well and the cells were incubated at 37°C for 5 h. The generated crystals were dissolved by dimethyl sulfoxide (DMSO) (Sigma-Aldrich) and absorbance was measured at 570 nm. The cell viability was normalized to that of untreated control cells.

### RNA isolation and quantitative reverse transcription-PCR (qRT-PCR)

Total cell RNA, vsRNAs, or supernatant viral RNA were extracted using RNAiso Plus (TaKaRa) and then reverse transcribed using the Primescript RT reagent Kit (TaKaRa) according to the manufacturer's protocol. Quantitative PCR (qPCR) was performed using the StepOnePlus^®^ Real-Time PCR System (Applied Biosystems) using FastStart Universal SYBR green master (Roche) and forward and reverse primers for PRRSV and cellular genes, as described previously [19]. Unless otherwise specified, RNA levels within each treatment group were normalized to HPRT1 mRNA as an internal standard (for primer sequence, refer to Table 2).

**Table 2:** The siRNA sequence and primers used for qRT-PCR

Primer name	Sequence(5'-3′)
vsRNA1-RT-Primer	CTCAACTGGTGTCGTGGAGTCGGCAATTCAGTTGAGACGTGTTG
vsRNA1-Stem-Loop-F	ACACTCCAGCTGGGTGCTGACTTGGCGC
Stem-Loop-R	CTCAACTGGTGTCGTGGAGTCGGCAATTCAG
let-7f-RT-Primer	CTCAACTGGTGTCGTGGAGTCGGCAATTCAGTTGAGAACTATA
let-7f-Stem-Loop-F	ACACTCCAGCTGGGTGAGGTAGTAGATTGTA
let-7f-Stem-Loop-R	CTCAACTGGTGTCGTGGAGTCGGCAATTCAG
U6-RT-Primer	CTCAACTGGTGTCGTGGAGTCGGCAATTCAGTTGAGAAAAATATGG
U6-Stem-Loop-F	CTCGCTTCGGCAGCACA
U6-R	AACGCTTCACGAATTTGCGT
PRRSV-ORF7- F	AGATCATCGCCCAACAAAAC
PRRSV-ORF7- R	GACACAATTGCCGCTCACTA
PRRSV-NSP2-F	TGAGTGAGCCCGTACTTGTG
PRRSV-NSP2-R	GATGCCCATGTTCTGCGATG
HPRT1-F	TGGAAAGAATGTCTTGATTGTTGAAG
HPRT1-R	ATCTTTGGATTATGCTGCTTGACC
Drosha-siRNA	CACAUGCGGAAGAAAGGGAU
Dicer-siRNA	UGAGAAGCAAAAAGGUCAGC
Ago2-siRNA1	AAGGAUAUGCCUUCAAGCCUC
RNase L-siRNA	CAGGAAGUCAAGAGAGAUCUAUU
NC-siRNA	UGCACUGUGCAAGCCUCUU
Drosha-qF	GTGCTGTCCATGCACCAGATT
Drosha-qR	TGCATAACTCAACTGTGCAGG
Dicer-qF	GCCATGGCAACAAGAAGCAA
Dicer-qR	TCTGTTGAAGTCTCCCCTGA
Ago2-qF	CAGTGCGTGCAGATGAAGAA
Ago2-qR	AGAAAGATGACGGGCTGCTG
RNasel-qF	GCTCAAAGTAATGAAGAGGT
RNasel-qR	ACATTCCGAAGCGTCCTA
β-actin-F	TCCCTGGAGAAGAGCTACGA
β-actin-R	AGCACTGTGTTGGCGTACAG

To determine supernatant PRRSV RNA copy number, standard curves were generated using serial dilutions of standard plasmid DNA bearing a 372 bp fragment of the PRRSV ORF7 sequence. RNA absolute quantities were calculated by normalization to the standard curve.

To analyze PRRSV-vsRNA1 expression, a highly sensitive and specific stem-loop real-time PCR approach was used for measurement of PRRSV-vsRNA1 expression as described previously, with minor modifications [51, 52]. Briefly, total RNA was reverse transcribed using specific primers for PRRSV-vsRNA1. The qRT-PCR reaction was performed using FastStart Universal SYBR green master (Roche) with the specific forward primers for PRRSV-vsRNA1 (listed in Table 2) and the universal reverse primer. RNA values were normalized to U6 small nuclear RNA as an internal reference.

To determine the absolute copy number of PRRSV-vsRNA, standard curves were calculated using the linear range within the dilution series of purified RNA oligonucleotides corresponding to PRRSV-vsRNA1 sequences (GenePharma). The copy number was calculated using the oligo concentration and the molecular weight of the transcript. The synthetic RNA input ranged from 75 copies to 7.5 × 10^8^ copies per reaction and the reaction products were analyzed using the SYBR green master and the StepOnePlus^®^ real-time PCR system as described above. RNA was extracted from various numbers of cells after quantitating the cells by counting them under a microscope. Raw cycle threshold (C_T_) values for each sample reaction were converted to absolute copy number/cell based on the curve.

### Northern blotting of PRRSV-vsRNA1

Northern blotting experiments were performed as previously described [53]. Briefly, total RNA was extracted from uninfected and PRRSV-infected cells (GD-HD, MOI = 1) using RNAiso Plus (TaKaRa) according to the manufacturer's instructions. Next, 10 ul of RNA was separated on a 15% polyacrylamide gel containing 8 M urea and transferred onto a nylon membrane (GE Healthcare) by electroblotting, then fixed to the membrane by ultraviolet cross-linking. Synthesized oligonucleotides complementary to the PRRSV-vsRNA1 were labeled with digoxigenin using the DIG Oligonucleotide Tailing Kit, 2nd Generation (Roche). The membranes were hybridized overnight at 42°C in hybridization solution. After hybridization and high stringency washes, signal detection was performed using the DIG Luminescent Detection Kit (Roche) according to the manufacturer's protocol. RNA oligomers of lengths of 21 to 38 nt were used as size markers.

### Viral small RNA isolation and cloning

Total RNA was isolated from PRRSV-infected PAM cells (GD-HD, MOI = 1) at 12 h post-infection (hpi) using RNAiso Plus (TaKaRa). RT-PCR was performed using stem-loop RT primers for PRRSV-vsRNA1. The RT-PCR products were cloned into pMD-18T (TaKaRa) and sequenced.

### Flow cytometry assay

MARC-145 cells were seeded into 24-well plates containing DMEM supplemented with 10% FBS at a density of 1 × 10^5^ cells/well. Twenty-four hours later, cells were infected with either rHPPRRSV/SD16/EGFP or rPRRSV/Ch-1R/DsRed (MOI = 0.01) in the presence of PRRSV-vsRNA1 or NC mimics (100 nM). The optimal signal observed for the cells infected with EGFP- or DsRed-tagged PRRSV was observed at 48 hpi. EGFP-PRRSV or DsRed-PRRSV-positive cells were detected by flow cytometry as described previously [50].

### Western blot analysis

To detect the expression of PRRSV structural protein N, PAM cells were transfected with PRRSV-vsRNA1 mimics or NC mimics and then infected with PRRSV. Western blotting was then performed as described previously [50] with minor modifications. Briefly, PAM cells were lysed and cellular proteins were separated on 12.5% SDS-PAGE gels and then transferred onto polyvinylidene fluoride (PVDF) membranes. After blocking with 5% milk in PBS containing 0.025% Tween-20 (PBST), the membranes were probed with mouse anti-PRRSV N protein monoclonal antibody (6D10) (1:1000) [54] or anti-α-tubulin antibody (1:5000) (Abcam). Primary antibody incubations were followed by incubation in HRP-conjugated anti-mouse IgG (Jackson Laboratories) as the secondary antibody. Immunostained proteins were visualized using ECL reagent (Pierce) according to the manufacturer's instructions.

### Virus titration

Virus progeny production was determined by titration as described previously [55], with minor modifications. MARC-145 cells were seeded into 96-well plates 1 day before virus infection. 100 μl of serial dilutions of virus supernatants were added to each well in replicates of eight. Cytopathic effect (CPE) was observed by microscopy after 2-3 days. Six days after infection, the 50% tissue culture infectious dose (TCID_50_) was determined using the Reed-Muench method.

### RNAi-mediated silencing of cellular genes

To knock down expression of cellular genes associated with miRNA biogenesis pathways, specific siRNAs against the Drosha/Dicer/Ago2/RNase L and a nonspecific NC siRNA were chemically synthesized with 2' OME modification (GenePharma). MARC-145 cells were transiently transfected with 50 nmol siRNA against Dicer/Drosha/Ago2/RNase L mRNA or NC siRNA using X-tremeGENE siRNA Transfection Reagent (Roche) according to the manufacturer's instructions. After transfection, cells were harvested for RNA quantification analysis (the siRNA and primer sequences are listed in Table 2).

### Computational prediction of vsRNAs structures and binding sites

The minimum free energy (MFE) and free energy of the thermodynamic ensemble of intermediate precursors giving rise to PRRSV-vsRNAs were predicted using online software tools RNAfold and MiPred. The putative mRNA targets for PRRSV-vsRNA1 within the PRRSV genome were predicted utilizing the computational tools RNAhybrid [56], TargetScan [57], and ViTa [58].

### Construction of psiCheck2 target luciferase reporters

The predicted binding site for PRRSV-vsRNA1 within the PRRSV genome was amplified by PCR using the primers listed in Table 3. The PCR product was cloned into the psiCheck2 vector (Promega). To verify the putative PRRSV-vsRNA1 target site, miRNA target site mutants were created. Two separate target site mutations were individually introduced into the seed region sequence and these mutated sequences were cloned into the psiCheck2 vector using site-directed mutagenesis to create psiCheck2-1666-MUT and psiCheck2-3249-MUT, corresponding to target sites 1 and 2, respectively.

**Table 3:** Mimics sequence and primer used for psiCheck2 vector construction

Name	Sequence(5'-3′)
PRRSV-vsRNA1-mimics	UGCUGACUUGGCGCAACACGU
PRRSV-vsRNA1-MUT-mimics	UCAUAGUCUCUCGCAACACGU
NC-mimics PRRSV-vsRNA1-inhibitor NC-inhibitor	AGCUGAUUUCGUCUUGGUA ACGUGUUGCGCCAAGUCAGCA UCACCGGGUGUAAAUCAGCUUGAC
psiCheck2-nsp1α-F	CCGCTCGAGATGTCTGGGATACTTGATCGGTGCAC
psiCheck2-nsp1α-R	TTGCGGCCGCTCTGCGGGAGCGGCAAGTTGGTTAAC
psiCheck2-nsp2-F	GCGATCGCGCCGGAAAGAGAGCAAGGAAAACACG
psiCheck2-nsp2-R	GGGTTTAAACCCGCCCAGTAACCTGCCAAGAATGGCAA
psiCheck2-nsp3-F	CCGCTCGAGGGGGCACGCTACATCTGGCACTTT
psiCheck2-nsp3-R	TTGCGGCCGCTCTCAAGGAGGGACCCGAGCTGAGA
psiCheck2-nsp9-F psiCheck2-nsp9-R	CCGCTCGAGGGAGCAGTGTTTAAACTGCTAGCCGC TTGCGGCCGCTCTCATGATTGGACCTGAGTTTTTCCC
psiCheck2-nsp10-F	CCGCTCGAGGGGAAGAAGTCCAGAATGTGCGGGTA
psiCheck2-nsp10-R	TTGCGGCCGCTTTCCAGGTCTGCGCAAATAGCGCGGA
psiCheck2-PRRSV-1666-WT-F	TCGAGGACAGGAACGGCGCTTGCGGTAGCGCCAAGTACGTGCTTAAACTGGAGGGTGGC
psiCheck2- PRRSV-1666-WT-R	GGCCGCCACCCTCCAGTTTAAGCACGTACTTGGCGCTACCGCAAGCGCCGTTCCTGTCC
psiCheck2- PRRSV-3249-WT-F	TCGAGATCATGCGTGAGGCTTGTGATGCGTCCAAGCTTAGTGATCCTGCTACGCAGGGC
psiCheck2- PRRSV-3249-WT-R	GGCCGCCCTGCGTAGCAGGATCACTAAGCTTGGACGCATCACAAGCCTCACGCATGAC
psiCheck2- PRRSV-1666-MUT-F	TCGAGGACAGGAACGGCGCTTGGCGAGAGACTATGAACGTGCTTAAACTGGAGGGTGGC
psiCheck2- PRRSV-1666-MUT-R	GGCCGCCACCCTCCAGTTTAAGCACGTTCATAGTCTCTCGCCAAGCGCCGTTCCTGTC
psiCheck2- PRRSV-3249-MUT-F	TCGAGATCATGCGTGAGGCTTGTGATGCGTAGAGACTATGAGATCCTGCTACGCAGGGC
psiCheck2- PRRSV-3249-MUT-R	GGCCGCCCTGCGTAGCAGGATCTCATAGTCTCTACGCATCACAAGCCTCACGCATGATC

### Luciferase assay

The luciferase reporter assay was performed as previously described [19] with the following modifications. HEK293FT cells (5 × 10^4^ cells/well) were seeded into a 48-well plate containing DMEM supplemented with 10% FBS. After 24 h, cells were co-transfected with reporter constructs containing predicted binding regions for PRRSV-vsRNA1: psiCheck2-nsp1α, psiCheck2-nsp2, psiCheck2-nsp3, psiCheck2-nsp9, psiCheck2-nsp10, psiCheck2-Target1-2-WT, or psiCheck2-Target1-2-MUT plasmids (50 ng), and the 100 nM of PRRSV-vsRNA1 mimics, PRRSV-vsRNA1-MUT mimics, or NC mimics using X-tremeGENE siRNA Transfection Reagent (Roche). Thirty-six hours post-transfection cells were lysed and luciferase expression was measured using a Synergy HT Multi-Mode Microplate Reader (BioTek, Winooski, VT) using the dual-luciferase reporter assay system (Promega) according to the manufacturer's instructions.

### Ago2-IP

PAM cells were mock-infected or infected with the GD-HD strain at an MOI of 0.1. Twelve hours later, RNAs in the lysates from PRRSV-infected or non-infected PAM cells were immunoprecipitated with anti-Ago2 monoclonal antibody (clone 2E12-1C9, Abnova) at 4°C for 4 h. Monoclonal anti-FLAG antibody (clone M2, Sigma-Aldrich) was used as an isotype control (IgG). The co-precipitated RNAs were purified from the lysates after treatment with DNase and Proteinase K. The PRRSV-vsRNA1 and NSP2-coding sequence of PRRSV genome RNA in the co-precipitate were detected using qRT-PCR.

### Statistical analysis

All experiments were performed with at least three independent replicates and representative data from all experiments are shown or expressed as the mean ± SD of independent experiments. Statistical significance was determined by Student's *t-test* when only two groups were compared or by one-way analysis of variance (ANOVA) when more than two groups were compared. A *P* value <0.05 was considered statistically significant.

## SUPPLEMENTARY FIGURES


