# Supplementary material for: MicroRNA-like viral small RNA from porcine reproductive and respiratory syndrome virus negatively regulates viral replication by targeting the viral nonstructural protein 2
Source: Oncotarget. 2016 Oct 17;7(50):82902–20. doi: 10.18632/oncotarget.12703 (PMC5347740; doi:10.18632/oncotarget.12703)
Supplement: Supplementary file 1 [file oncotarget-07-82902-s001.pdf]

MicroRNA-like viral small RNA from porcine reproductive and respiratory syndrome virus negatively regulates viral replication by targeting the viral nonstructural protein 2

SUPPLEMENTARY FIGURES

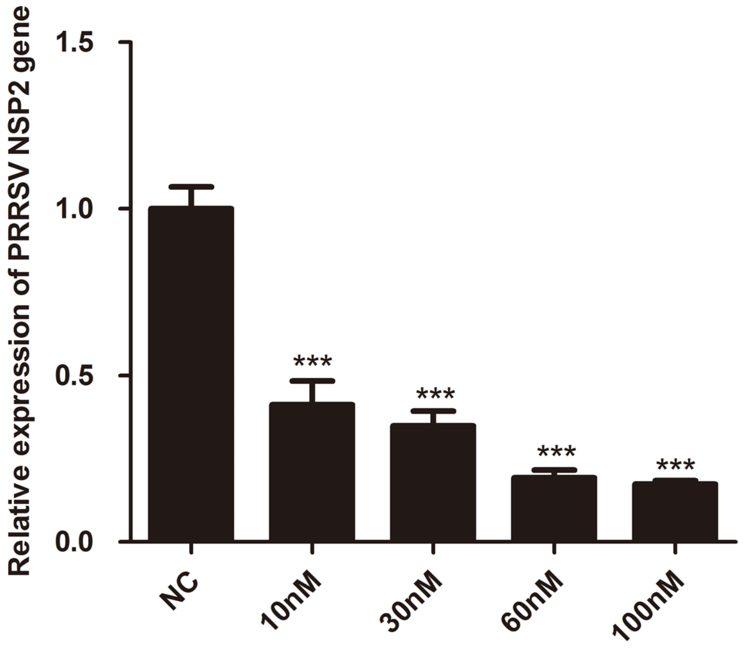

Supplementary Figure S1: PRRSV-vsRNA1 inhibits PRRSV replication in a dose-dependent manner. PAMs were transfected with different concentrations of PRRSV-vsRNA1 mimics followed by infection with GD-HD (MOI = 0.01) for 24 h. The expression levels of PRRSV NSP2 gene were assayed by qRT-PCR. Results are expressed as mean ± SD of three independent experiments. ***$p < 0.001$.

Majority	--- TTGTGATGCGTCCAAGCTTAGTG ---	
	10 20	Accession
GD-HD.seq	--- TTGTGATGCGTCCAAGCTTAGTG ---	KP793736.1
GD3.seq	--- TTGTGATGCGTCCAAGCTTAGTG ---	GU269541.1
HB-1-3.9.seq	--- TTGTGATGCGTCCAAGCTTAGTG ---	EU360130.1
HEB1.seq	--- TTGTGATGCGTCCAAGCTTAGTG ---	EF112447.1
Henan-1.seq	--- TTGTGATGCGTCCAAGCTTAGTG ---	EU200962.1
HUB2.seq	--- TTGTGATGCGTCCAAGCTTAGTG ---	EF112446.1
HUN4.seq	--- TTGTGATGCGTCCAAGCTTGGTG ---	EF635006.1
Jiangxi-3.seq	--- TTGTGATGCGTCCAAGCTTAGTG ---	EU200961.1
JXA1.seq	--- TTGTGATGCGTCCAAGCTTGGTG ---	EF112445.1
JX143.seq	--- TTGTGATGCGTCCAAGCTTGGTG ---	EU708726.1
SD16.seq	--- TTGTGATGCGTCCAAGCTTAGTG ---	JX087437.1
SX-1.seq	--- TTGTGATGCGTCCAAGCTTAGTG ---	GQ857656.1
SX2007.seq	--- TTGTGATGCGTCCAAGCTTAGTG ---	EU880434.2
SX2009.seq	--- TTGTGATGCGTCCAAGCTTAGTG ---	FJ895329.1
SY0608.seq	--- TTGTGATGCGTCCAAGCTTAGTG ---	EU144079.1
VR2332.seq	--- ATGTGATGCGACTAAGCTTGATG ---	EF536003.1
BJ-4.seq	--- ATGTGATGCGACTAAGCTTGATG ---	AF331831.1
CH-1a.seq	--- TTGTGATGCGGCTAAGCTTAGTG ---	AY032626.1
CH-1R.seq	--- TTGTGATGCGGCTAAGCTTAGTG ---	EU807840.1

Supplementary Figure S2: Sequence homology of PRRSV-vsRNA1 target sequence for the genotype II PRRSV strains. The accession numbers for the PRRSV strains are listed.
